# Assessment of community-based flood early warning system in Malawi

**DOI:** 10.4102/jamba.v14i1.1166

**Published:** 2022-03-31

**Authors:** Dickson D. Chinguwo, Dorothea Deus

**Affiliations:** 1 Department of Geospatial Sciences and Technology, The School of Earth Sciences, Real Estate Studies, Business and Informatics (SERBI), Ardhi University, Dar es Salaam, Tanzania; 2 Department of Computing and Information Technology, Faculty of Applied Science, The Malawi Polytechnic, University of Malawi, Blantyre, Malawi

**Keywords:** early warning system, community based early warning system, civil protection committee, river gauge, floods

## Abstract

One of the major natural hazards the world is facing these days are floods. Malawi has not been spared. Floods have affected the countries’ socio-economic developmental plans. River gauges have been installed along major rivers to monitor water levels in a bid to warn communities of imminent flooding. In Malawi, ever since the installation of river gauges no study has been done to assess their effectiveness. This study examines the effectiveness of these river gauges as part of community-based early warning system. The research employs both qualitative and quantitative approach. Questionnaires, interviews, group discussions, document analysis were all used in order to understand the behavioural aspect of communities under study. The current community-based early warning system practices were benchmarked against the following elements: risk knowledge, technical monitoring and warning services, dissemination and communication of warnings and response capability. The study revealed that Malawi has two distinct systems in place: at national level (managed by several government departments) and at community level [managed by Civil Protection Committees (CPCs)]. These systems were installed by non-governmental organisations (NGOs) and faith-based organisations. Apparently, no direct link exists between the two. Operational bureaucracy affects the speedy presentation of warning messages at national level. Lack of capacity and necessities affects the operation of the community-based system. Despite the efforts to develop the early warning systems, the failures outweigh the successes. Government needs to provide enough funding for systems sustainability, build capacity of CPCs and install more technologically advanced systems.

## Introduction

The world at large is affected by a number of natural hazards: these include dry spells, earthquakes, wild fire, floods, strong winds, hurricanes just to mention a few. One of the major hazards affecting most countries is floods. Flooding affects heavily the socio-economic aspects of most communities and countries at large. In Malawi floods have also affected infrastructure such as roads, bridges, buildings and farm lands (GoM 2015c, 2019; Pauw et al. 2010; Pauw & Thurlow 2009). It is estimated that US$9 million or 0.7% of Gross Domestic Product (GDP) is lost each year in the southern region of Malawi because of floods (Pauw & Thurlow 2009).

Like most of the countries around the world, Malawi also faces a number of natural and man-made disasters, which includes floods, drought, earthquakes, diseases outbreaks, strong winds, fire and accidents just to mention a few (Hagenlocher et al. 2012; MacOpiyo 2017). Over the years, the intensity and frequency of disasters have been increasing and affecting the livelihood of people physically, socially and economically (GoM 2015b). According to the Global Facility for Disaster Reduction and Recovery (GFDRR) (2019) report on the disaster risk profile for Malawi, drought and floods top the list on major disasters affecting the country. Recently, floods have had a devastating impact on the social and economic environment of the country.

The greatest threat to the people, property and economy of Malawi are natural hazards. Since 1946, of all the 298 times the country has been impacted by hazards, 89% of those have been natural hazards and the rest man made (Misomali 2014). Records indicate that in the last 100 years, the country has experienced about 20 droughts, eight of them in the last 36 years and affected over 24 million people (MacOpiyo 2017). In 2015, for example, Malawi experienced a once-in-500-year flood, which impacted the country socially and economically (MacOpiyo 2017). During this time, Malawi suffered a property loss of about US$335m with recovery and reconstruction cost of about US494mn, about 1.1 million people were affected by the floods with 230 000 people displaced, 173 missing and 106 killed (GoM 2015c; MacOpiyo 2017).

Efforts to monitor flood based on community early warning system were initialised. The first river gauge installed in Malawi was along Shire River at Liwonde barrage in 1948 (Sene et al. 2017). The reasons for the installation were to help in the operation of the barrage and management of the hydropower and irrigation schemes further downstream (Sene et al. 2017). From around 2007, the Disaster Preparedness European Civil Protection and Humanitarian Aid (ECHO) programme (DiPECHO) under the Director-General for European Civil Protection and Humanitarian Aid Operations (DG ECHO) have worked with a number of non-governmental organisations (NGOs) such as Cooperazione Internazionale (COOPI), Evangelical Association of Malawi (EAM) and Goal Malawi to install river gauges in some notable rivers (Lingadzi, Likangala, Shire), which flood almost yearly (De Goyet et al. 2011; Hagenlocher et al. 2012; Stepanyan 2014).

**FIGURE 1:**
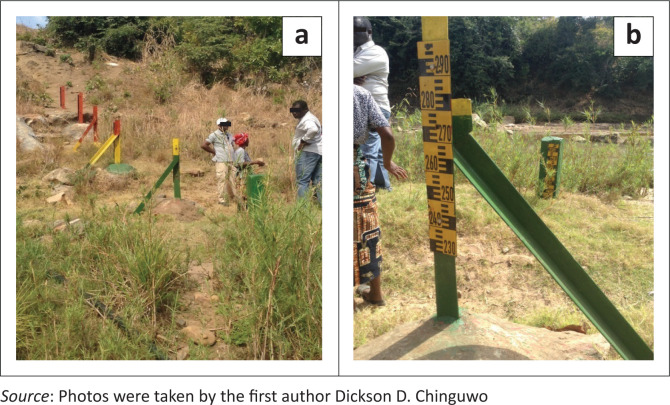
(a) Cross section of river gauges, (b) river gauge with readings. Location, Zomba District.

By deduction, most of the Community Based Flood Early Warning System (CBFEWS) within the communities come in the form of steel bars erected in rivers at a vantage point, where accurate readings of the river water levels can be obtained. The steel bars are colour coded (green, yellow and red – refer Table 1 for colour code meaning) against appropriate levels indicating the depth of water levels – how much it has risen or receded from the river bed, Figure 1(a). The readings in Figure 1(b) indicate the depth of water in metres (m) from the river bed.

**TABLE 1:** River gauge colour sode meaning.

Colour code	Alert meaning/level
Green	The water levels are normal, no warning message to be discharged
Yellow	Intermittent danger of water flooding, communities they must prepare for evacuation to safety grounds
Red	Dangerous level, communities have to evacuate to safety grounds or evacuation centres

The sole purpose of erecting the river gauges is to monitor the rising and falling levels of rivers in selected areas as an early warning system. This has been explained more in the study’s discussions section. The installation of river gauges supposedly led to the institutionalisation of river-line alliances between villages where the gauges were installed.

The problem is that ever since the installation of the community-based early warning systems (CBEWS) no assessment has been done to assess its effectiveness. Thus the objective of this study is to assess whether the installed river gauges used in CBEWS have been effective in preventing catastrophic disasters in loss of life or loss of property. The rationale behind the study is to assess whether there is need to improve on the current manual system of the community-based early warning for floods.

## Literature review

### Early warning system in Malawi

United Nations International Strategy for Disaster Reduction (UNISDR) defines early warning systems (EWS) as ‘an interrelated set of hazard warning, risk assessment, communication and preparedness activities that enable individuals, communities, businesses and others to take timely action to reduce their risks’ (UNISDR 2015). Responding to the Hyogo Framework for Action priority area 2 on knowing the risk and taking action, the government of Malawi instituted the development of EWS through its National Disaster Risk Management Policy of 2015 (GoM 2015b). Priority areas 2 and 3 called for the establishment of a comprehensive system for disaster risk identification, assessment and monitoring, and development and strengthening of a people centred early warning system, respectively (GoM 2015b) and finally the Malawi National Flood Early Warning System (F-EWS) was established.

**FIGURE 2:**
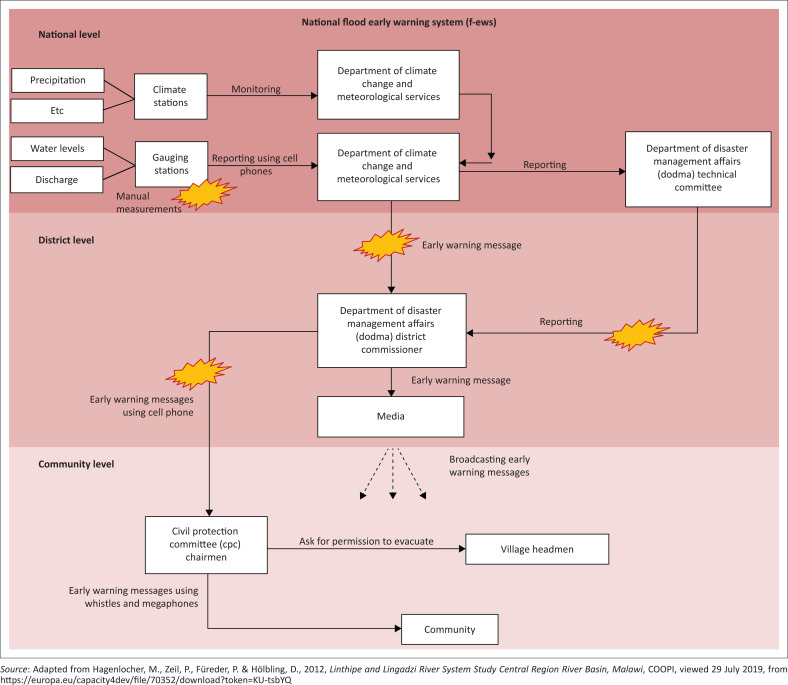
National flood early warning system.

Unfortunately, the Malawi F-EWS is not discussed much in literature. Figure 2 summarises how the F-EWS is set up. It is divided into three levels of operation: national level, district level and community level. It is reported that F-EWS is primarily run and managed by Department of Climate Change and Metrological Services (DCCMS), Department of Water Resources (DWRs) and Department of Disaster Management Affairs (DoDMA) (GoM 2015b). Department of Climate Change and Metrological Services and DWR have a number of gauging stations on major rivers that are used to measure water levels and discharge. Despite its strength of having a number of gauging station in some major rivers plus a strong institutional link between involved departments, F-EWS has a number of challenges too. Since there are not enough gauging stations and a lot of bureaucracy in communications, there is no guarantee that communities get the early warning messages in time, also no models are used to predict floods, just to mention a few (GoM 2015b).

### Community-based early warning system

Within local communities, EWS are sometimes referred to as CBEWS (Smith, Brown & Dugar 2017) or people-centered early warning systems (PCEWS) (UNISDR 2006). Therefore, CBEWS for floods are there to help local communities in using available resources to efficiently prepare and respond to floods event (Smith et al. 2017). For an effective and efficient CBEWS, UNISDR (2006) developed a checklist comprising four crucial elements (Table 2) in instituting EWS. Almost all the EWS are developed around these elements.

**TABLE 2a:** Four elements for developing a community-based early warning system.

Risk knowledge	Monitoring and warning service
**Systematically collect data and undertake risk assessment** Are the hazards and the vulnerabilities well known? What are the patterns and trends in these factors? Are risk maps and data widely available?	**Develop hazard monitoring and early warning services** Are the right parameters being monitored? Is there a sound scientific basis for making forecasts? Can accurate and timely warnings be generated?

*Source*: UNISDR, 2006, ‘Developing early warning systems: A checklist’, in *Third International conference on early warning*, Bonn, Germany, viewed from https://www.unisdr.org/files/608_10340.pdf.

**TABLE 2b:** Four elements for developing a community-based early warning system.

Dissemination and communication	Response capability
**Communicate risk information and early warnings** Do warnings reach all of those at risk? Are the risks and warnings understood? Is the warning information clear and useable?	**Build national and community response capabilities** Are response plans up to date and tested? Are local capacities and knowledge made use of? Are people prepared and ready to warnings?

*Source*: UNISDR, 2006, ‘Developing early warning systems: A checklist’, in *Third International conference on early warning*, Bonn, Germany, viewed from https://www.unisdr.org/files/608_10340.pdf.

The four elements are the cornerstones used to benchmark the effectiveness and efficiency of CBEWS. Risk knowledge is measured by the community’s ability of knowing and understanding the kind of vulnerability that they are prone to. Technical monitoring and warning services stipulate that the vulnerable community must have the ability and capability of monitoring the hazard and producing warning messages. Dissemination and communication of warning promotes that generated warning messages have to be clear enough and should be able to reach out the community at risk. Molinari et al. (2015) also observed that during emergencies, there is need to increase the level of communication by improving the target warning messages. Response capability is the capability of the community to take heed to the warning messages, the community’s ability to react to the warnings and take appropriate action. Khan et al. (2018) used the same benchmark in studying the effectiveness of CBEWS in the State of Azad Jammu and Kashmir. Dugar et al. (2017) and Brown and Dugar (2018) also used the four elements as benchmark to establish the effectiveness of CBEWS in Nepal by looking at the lead time.

**FIGURE 3:**
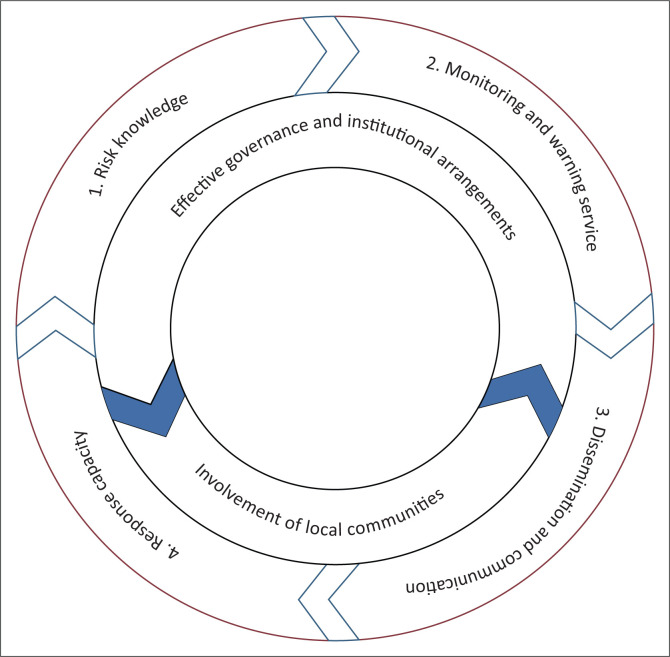
Conceptual framework for assessing community-based early warning systems.

In addition to these elements, there are other important issues to be considered in developing CBEWS. These include issues on effective governance and institutional arrangements, involvement of local communities, consideration of gender perspectives and cultural diversity and a multi-hazard approach on EWS (UNISDR 2006). In view of these difficulties a conceptual framework (Figure 3) was developed as a benchmark for assessing CBEWS in Malawi.

## Materials and methods

### Study area

**FIGURE 4:**
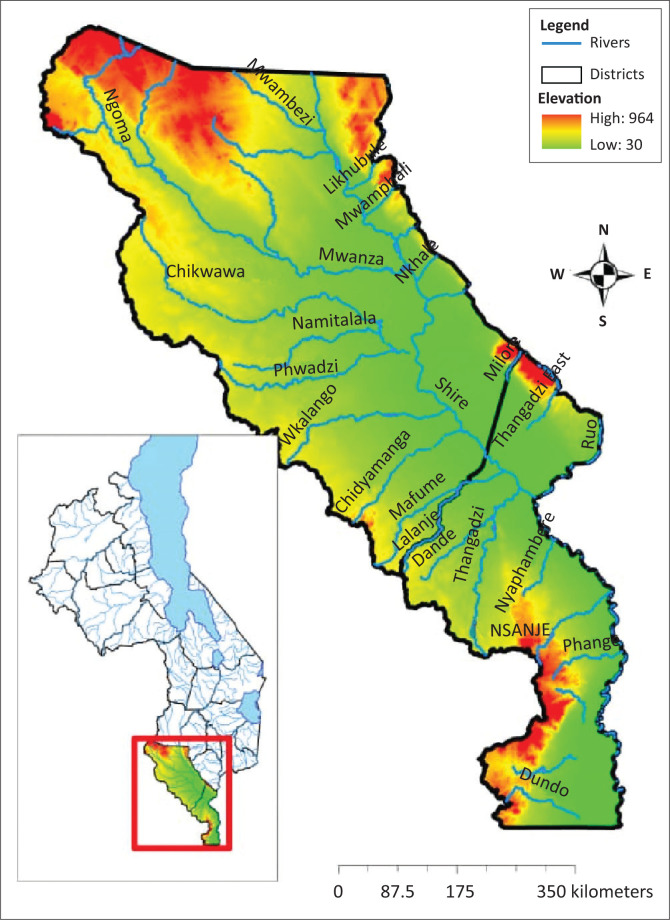
Map showing study areas.

The study was conducted in two districts (Figure 4) in the southern region of Malawi: Nsanje and Chikwawa. Even though these are the main areas concerned, some other districts within the country were visited for a comprehensive and richer understanding of the status of EWS within the country. The other districts are Salima and Dedza (in Central Region), which are along the lakeshore of Lake Malawi and Zomba and Phalombe (in Southern Region). These two districts lie at the base of Zomba and Mulanje mountains, respectively.

Nsanje and Chikwawa districts were selected for the study because geographically they are located in the low lying areas and the major river Shire river runs through these districts. According to the Malawi disaster profile on floods (GFDRR 2019; GoM 2019) was based on the intensity and frequency of occurrence of floods in those districts.

In order to have a holistic view and clear understanding of the workability and performance of the CBEWS in Malawi, the study employed a mixed (qualitative and quantitative) approach type of research. Qualitative method is mostly used in behavioural sciences where the researcher attempts to draw meanings out of natural phenomenon. On the other hand, quantitative method is mostly used in ‘hard science’. Quantitative methods assume a fixed and measurable reality, which is concerned with discovering facts about a phenomenon (Jonker & Pennink 2010). Therefore, data collection for this research was done through questionnaires, interviews and group discussions and document analysis.

### Document analysis

The term document extends beyond just written materials and includes any symbolic representation that can be recorded and retrieved for analysis (Oates 2006). In this regard documents include textual documents (e.g. reports, memos and academic publications), audiovisual data (e.g. photographs, diagrams, animations, video and sounds) and electronic data (e.g. screenshots, computer games and archives) (Oates 2006).

During the study, a wide range of documents related to disaster management in Malawi were collected and reviewed. Such documents amongst others included policy documents – to gain insight into what the policy states on managing a disaster; what has been reported on the EWS; in Malawi; assessment forms that indicate what information is collected after the disaster and research publications to understand what has been done already relating to the study and identify the gaps within the study areas. Some of the documents that were reviewed included: Disaster Risk Management Operational Guidelines for the Republic of Malawi; A Disaster Rapid Assessment Form; Disaster Impact and Needs Reporting Form and The National Disaster Risk Management Policy.

### Interviews and group discussions

For a better and clear understanding of what really happens when a disaster happens, group discussions were conducted within the selected communities and non-probability purposive sampling was used. This sampling technique ‘does not need underlying theories or a set number of key informants’ (Tongco 2007). The researcher decides on whom to interview on the basis of their experience and knowledge in the particular field (Creswell & Creswell 2017; Tongco 2007). The key informants (KIs) in this research were those members who know much of the phenomenon and are proper representation of the entire population. Participants or informants included members of District Civil Protection Committee (DCPC), Village Civil Protection Committee (VCPC) and Areal Civil Protection Committee (ACPC). Members of the committees are from government agencies, departments and NGOs. A total of nine Group Village Heads (GVHs) were visited from six districts of Salima, Zomba, Phalombe, Nsanje, Chikwawa and Dedza, respectively. In each of the GVHs, two VCPCs and two ACPCs formed the grouping of KIs with whom interviews and discussions were carried out. On average the discussions and interviews took about 60 min. Predefined questions were used. Depending on the answers provided, further questions were asked seeking for further clarification. The sessions were recorded so as to go through the material at a later stage for any missing information.

### Questionnaires

As a result of constraint in time and busy schedules of prospective respondents, questionnaires were also used to collect data from three key staff from DoDMA and a total of 37 members of DCPC: 16 members from Nsanje and 21 members from Chikwawa Districts. Of the 37 members, 81% were male and 19% were female. A total of 27% were between ages 25 and 30 years, 16% between 31 and 35 years, 33% between 36 and 45 years and 24% were above 45 years of age. This grouping consists of officers from different government departments and NGOs. There is a deliberate move that within the members there must be at least a female and a youth.

Some of the questions that were asked during the course of interviews and on the questionnaire include the following: (1) Are there any CBEWS installed within their villages? (2) How vibrant are their Civil Protection Committees (CPCs) in relation to warnings because of imminent flooding? (3) What challenges are being faced with the operations of the CBEWS? (4) Are the CBEWS being effective within their areas?

### Data analysis

This research assumed a mixed approach type of research. With this type of approach, analysis occurs both within the qualitative (description and thematic text or image analysis) approach and the quantitative (descriptive and inferential numeric analysis) approach (Creswell & Creswell 2017). Quantitatively, International Business Machines Corporation Statistical Package for the Social Sciences (IBM SPSS) Statistics was used in analysing responses from the questionnaire administered to DCPCs. Descriptive analysis and frequencies in Results and Discussion sections of certain responses were derived from this. Whilst MS Excel was used in tabulating graphs, providing comparative visualisation of response obtained from SPSS. This provided a clear understanding behind the meaning of the outcomes.

### Ethical considerations

This article followed all ethical standards of research without direct contact with human or animal subjects.

## Results

Qualitatively, data transformation strategy was used in this study: a strategy whereby a researcher quantifies qualitative data by creating codes and themes qualitatively, then counts the number of instances they occur in the responses given in counting lines or sentences (Creswell & Creswell 2017). Tables 3 and 4 present a brief illustration of the analytical process followed on the qualitative data collected. This is based on the following two questions: (1) what does the DCPC and DC office do when there is a river flooding within the district? (2) what should be done to improve on the current practices of the system?

**TABLE 3:** Activities taking place during flooding.

Extracts from responses	Theme
**‘[*C*]arry our needs assessment** and ….’	Conduct assessment
**‘[*M*]itigations to protect** people’s lives and safeguard property’	Mitigation measures
‘[*S*]end warning messages for people to **evacuate to safe places’**	Relocating people
‘DC and DCPC **calls for inter-cluster meeting’**	Emergency meetings
‘[*I*]nform DoDMA and other partners **to provide assistance’**	Solicit assistance
**‘[*S*]end warning messages** for people to evacuate to safe places’	Issue warnings
‘Conduct **search and rescue missions** if there is need to do so’	Search and rescue

DC, District Commission; DCPC, District Civil Protection Committee; DoDMA, Department of Disaster Management Affairs.

**TABLE 4:** Proposed improvements of the current system practices.

Extracts from responses	Theme
‘[*C*]ommunities **to be trained and own the system** to safeguard the system’	Capacity building
‘[*I*]mprove **funding and equipment’**	Funding
‘[*N*]eed to orient the operators now and again **to enable them to be updated on latest methods of conducting** CBFEWS’	Improve technology
‘CBFEWS should **be adequately placed; a number of systems** have to be provided for diversity’	Increase CBFEWS
‘[*T*]raining of communities in **making use of available resources’**	Available resource use

CBFEWS, Community Based Flood Early Warning System.

During the course of the research, a number of things transpired with regard to CBEWS for floods in the districts visited and Malawi as a whole.

For a better comprehension and assessment on the whole workability of CBEWS it is imperative to group the elements of Figure 3 into two: administrative and operations. Administrative looking at the management of the whole CBEWS whilst operations for the daily running of the CBEWS. Effective governance and institutional arrangement and the involvement of local communities fall under the administrative, whilst operations encompasses response capacity, dissemination and communications, monitoring and warning services. As for risk knowledge, this cuts across the two groups because there is need to be aware of risk knowledge.

### Arrangement set-up

Administratively, two distinct EWS have been fused together in an attempt to form a national F-EWS. At national level – this is run and managed by governments’ departments: DCCMS, DWR and DoDMA. At local levels, which are within communities prone to flood, they are managed by CPC. Members in some of the committees are volunteers or elected by members within the communities. Figure 2 summarises how the CBEWS are supposedly integrated with the F-EWS.

Technically, the system run by government departments, have weather monitoring stations whose information and data are sent to the offices for further processing. Communications of warnings are carried out after some bureaucratic procedure has taken place. Whilst at community level, it is the designated persons within the committee who are responsible for reading the water levels off the river gauges and disseminating information accordingly.

### Key elements of community-based early warning systems

#### Risk knowledge

It was reported during the group discussions, interviews and responses on questionnaires that communities are aware of the risks of floods in their areas. Each and every district has a hazard map that shows which parts of the district are prone to floods. This was cemented with responses such as ‘in some areas there are inter-village or inter-district alliances, which share information on floods’ which verifies that people are aware of the hazard risk. In Salima one response was (in Chichewa) ‘madzi ndi alendo sachedwa kupita’ meaning ‘flooding waters are like visitors, they don’t stay for long’. In Chikwawa and Nsanje, it was reported that when they notice swarm of ants or hippos moving from rivers it signifies a possibility of flooding that season.

#### Monitoring and warning services

Literature and reports show that in Malawi flood warning system exists. One respondent from DoDMA explained that the country has two distinct systems: at national level managed by DCCMS and DWR and at community level managed by CPC with the help of developmental partners or NGOs, although there are problems with this set-up. Since there is no direct linkage between the two systems, there is a lot of bureaucracy in getting the messages across (refer to Figure 2). Also the community based systems are mostly NGO or church based sponsored and are tied to projects, which have life spans. When the deadline of project expires, funding ceases. These projects are also prone to vandalism, which compromises the monitoring.

#### Response capability

At national, district or community levels, guidelines have been put in place to assist in response to flooding incidences. As stated by one Director of Planning and Development (DPD), periodical meetings are convened between CPCs (DCPC, VCPC, ACPC) to strategise, remind and reaffirm each other of response plans, evacuations plans and evacuation areas in case a flood hits.

#### Dissemination and communication

By the time of this research, about 98% of DCPC members ascertained that the mechanism to disseminate and communicate information is in place. The remaining 2% of DCPC are unaware because they are new within the districts. As pointed out in the previous section that CPCs have periodical meetings, some of these meetings serve as avenues of disseminating and communicating some vital information between DoDMA, DCs office and communities concerned. During floods, various means of communication are used. Table 5 gives the preferable means of communication during flood disasters.

**TABLE 5:** Response to common means of communication.

Preferred means	Response rate
Whistles	22
Community radio	21
Megaphones	17
Drums	12
Colour flags	12
Word of mouth	10

In places where community radios are unavailable, whistles are preferred. Whistles and megaphones are used mostly to warn people when floods are imminent.

### Strength Weaknesses Opportunities Threats analysis

**FIGURE 5:**
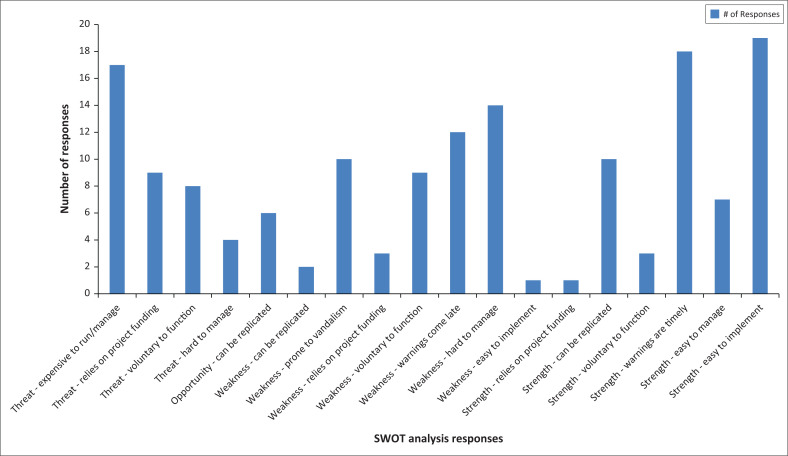
Community-based early warning systems SWOT analysis.

**FIGURE 6:**
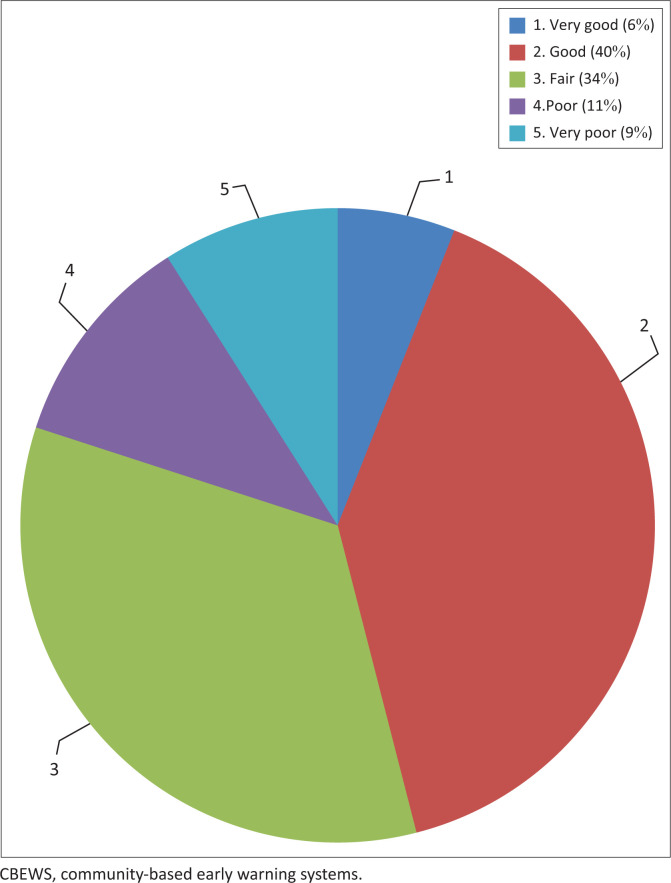
Performance rating of community-based early warning systems.

The respondents to the research were asked what are the strengths and weaknesses of the CBEWS, the opportunities the current CBEWS is presenting and threats the CBEWS is exposed to. Figure 5 presents the outcome of the Strength Weaknesses Opportunities Threats (SWOT) analysis based on the responses from the respondents. From the SWOT analysis it is obvious that 25% account to threats to the current system, 33% present opportunities posed by the systems whilst strengths amount to 38%. From all these findings, the current CBEWS presents 1% opportunity that it can be easily replicated. Figure 6 shows how the CBEWS are viewed by different member of CPCs.

## Discussions

In responding to Priority area 2 of the Sendai Framework for Disaster Risk Reduction 2013–2030, the Malawi Government instituted the National Disaster Risk Management Policy in which Priority area 3 of the policy (GoM 2015b) recommends that the country must develop and strengthen a people centred early warning system. People centred means the focus is on individuals, communities and organisations that are at risk. All these involved in the generation of early warning information, access this information in a timely manner, enabling them to act swiftly and in good time so as to minimise loss of life and goods.

### Risk knowledge

**FIGURE 7:**
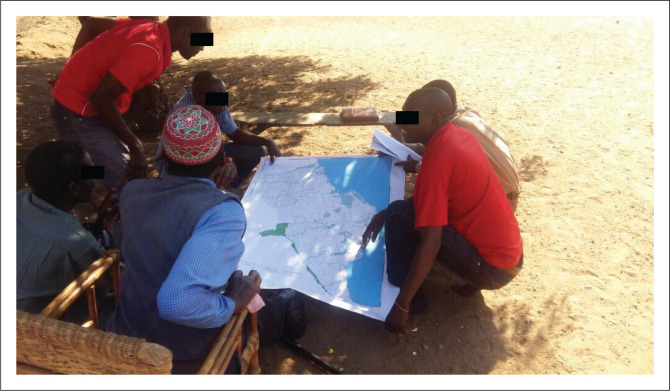
Sharing hazard map with community members.

Under the element of risk knowledge, the KIs highlighted that everyone within the affected areas within the district are aware of the prevailing hazards and vulnerabilities. In most of these areas floods re-occur on a yearly basis, the communities are very conversant with the dynamics of the hazard. Evacuation plans and routes are known to all courtesy of the periodic meetings community leaders call for to update and sensitise communities to the weather patterns. Hazard maps (Figure 7) showing flood prone areas are made available by DoDMA to DC and TA offices.

### Technical monitoring and warning services

From the interviews with members of the DCPCs, it was revealed that the villagers do not have the technical expertise and know-how to monitor hazards. The local communities only monitor the rising and falling of water levels in rivers using the river gauges (Figure 1). Apart from river gauges, weather monitoring stations in specific areas of the districts have been installed by the Ministry of Agriculture, the DWR and the DCCMS. There are stations to monitor weather patterns and help in predicting whether the weather will be favourable or not. Unfortunately, there is no direct linkage between these tools and CBEWS found in the districts (Hagenlocher et al. 2012).

### Communication and dissemination of warning messages

The essence of EWS is to save lives and loss of property. From the given findings, in all the districts it was reported that there are different means of communicating and disseminating warning message. These include community radios, megaphones, drums and word of mouth. It was reported that the warning messages are conveyed and communicated by the members of the VCPC and ACPC although these are mostly communicated when the danger is imminent (when water is at red mark on the river gauge). Megaphones, whistles and drums are used in warning people of imminent flooding so that they move to higher grounds or safer places. The bureaucratic setting the system (GoM 2015b; Hagenlocher et al. 2012; Mijoni & Izadkhah n.d.), is ideal for warnings whose time span is longer because the information trickles down to vulnerable communities late. ‘The other problem with this setting is that warning messages are generalised and not area specific, hence at times people don’t take them seriously’ – commented one respondent in Salima. The same sentiments were shared in Dedza, Zomba and Nsanje. Warning massages have to be specific to an area.

### Response capability

Within the Disaster Risk Management Operational Guidelines (GoM 2015a), local authorities have been mandated to ensure that their respective communities are capable of responding to disasters. Multi-hazard contingency plans, evacuation plans and recovery plans have to be developed by local authorities in line with national plans.

In all the places visited, KIs under the VCPC and DCPC stated that periodic meetings are held in respective villages, which are exposed to flood hazards reminding each other of what is expected of them during the disaster. Luckily enough each area has its own identifiable safe place when floods hit. School grounds, places of worship area, nearby hills have all been identified as safe havens when floods arise. Sometime drills are carried out simulating flood disaster and to test the response rate of the community (COOPI 2018).

### Challenges faced by the community-based early warning systems

If there is a weakness or failure in any element of a system, the whole system is bound to fail (Macherera & Chimbari 2016; Molinari et al. 2015). Although the four key elements were considered in CBEWS implementation in Malawi, the system is facing some challenges. ‘Lack of resources or funding’ supporting the operations of CBEWS (PARTICIP 2017) came up as one of the main challenges the systems is facing. Civil Protection Committees members not having mobile phones to use or even credit in the phones to alert others by calling or messaging also presents problems (Hagenlocher et al. 2012). Furthermore, most of the river gauges have been installed under projects that have life span (Hagenlocher et al. 2012). These are not sustained when the project concludes its operations such as the ones in Salima the project concluded in 2017. In addition, ‘lack of proper coordination’ amongst different stakeholders has greatly affected the operations of the CBEWS. ‘Absence of inter-river or inter-districts alliances’ in most districts has rendered information not being passed from upper-stream to down-stream communities.

Continual change of CPCs that has resulted in ‘deficiency in CPCs capacity’ to interpret and carry out duties properly is another factor in contributing to the collapse of the system in place. As the case in Nsanje where it was reported that most outgoing CPCs do not provide handover observed for the incoming ones. Chiusiwa lamented that there is need to build capacity of the communities to enable them to ably interpret and use early warning information effectively (Chiusiwa 2015). One of the major deterrents to the success of the CBEWS is that only major rivers are monitored whilst small rivers and other tributaries that equally contribute to flooding are not monitored (Chiusiwa 2015).

### Community-based early warning systems performance

With reference to Figures 5 and 6 on how the CBEWS are faring, it can be observed that the systems are not performing as anticipated. A total of 46% (sum of very good and good) respondents perceived that the CBEWS in place are helping and performing above average whilst 54% think otherwise. As stated by Van Belle, Eccles and Nash (2003) that within a system, there is a special relationship between components that are interrelated and are supposed to work together for a specific objective or purpose. Unfortunately, this is not the case with CBEWS in Malawi. Community-based early warning system are prone to vandalism, heavily relying on donor funding, managing them on voluntary basis and the fact that warnings come late are some of the aspects rendering the CBEWS ineffective. Macherera and Chimbari (2016) and Khan et al. (2018) concurred with the UNISDR that CBEWS are bound to fail if there is a failure or weakness in any element of that system.

## Conclusion

As one of the major natural hazards Malawi faces is floods, this study aimed at assessing the set-up and management of CBEWS for floods in the country. The analysis involved benchmarking the CBEWS against four key elements and some cross-cutting issues involved in the development of it thereof (UNISDR 2006). The elements in consideration were grouped into administrative, technical and operations. Through this study, it was observed that two separate EWS exist in Malawi – at national level (F-EWS) and at community level (CBEWS) F-EWS managed by DoDMA with inputs from DWR and DCCMS whilst CBEWS managed by CPCs in areas that are installed. Unfortunately, there is no direct link between the two systems.

Despite proper consideration on issues surrounding the developments of CBEWS, it has been discovered that operational bureaucracy posed a serious threat to the effective running of CBEWS. Heavy reliance on donor project funding that has limited life spans is one contributing factor. Another is vandalism of river gauges, which in turn has a bearing on the whole system as it becomes expensive to maintain and run. In addition, the community respondents recommended that there is need for continual trainings on CPCs in areas of interpreting and using early warning messages because the committees change from time to time. Another area of concern was on the number of gauging stations. Not all rivers that cause havoc when flooded are monitored as such communities wished if all the rivers were monitored.

The following are recommendations suggested by the respondent in order to improve the shortfalls of the CBEWS:

- Increase funding to sustain what is already there, as most of the CBEWS rely on project funds.
- Capacity building of CPCs to properly manage the systems in terms of interpretation and making use of early warning messages.
- Increase the number of monitoring stations to include other tributaries that cause floods.
- Strengthen the inter-river and inter-district alliances.
- Improve the technology used in monitoring floods. Locally available, modern and advanced technologies/equipment have to be used.
